# NRF2 in Cancer: Cross-Talk with Oncogenic Pathways and Involvement in Gammaherpesvirus-Driven Carcinogenesis

**DOI:** 10.3390/ijms24010595

**Published:** 2022-12-29

**Authors:** Mara Cirone, Gabriella D’Orazi

**Affiliations:** 1 Department of Experimental Medicine, University of Rome La Sapienza, Viale Regina Elena 324, 00161 Rome, Italy; 2 Department of Neurosciences, Imaging and Clinical Sciences, University “G. D’Annunzio”, 66013 Chieti, Italy; 3 School of Medicine, UniCamillus International University, 00131 Rome, Italy

**Keywords:** NRF2, p53, gammaherpesviruses, oxidative stress, reactive oxygen species (ROS), cancer therapy, chemoresistance, p62/SQSTM1, inflammation, KEAP1, p21, mTOR, NFkB, apoptosis, autophagy, STAT3

## Abstract

Expanding knowledge of the molecular mechanisms at the basis of tumor development, especially the cross-talk between oncogenic pathways, will possibly lead to better tailoring of anticancer therapies. Nuclear factor erythroid 2-related factor 2 (NRF2) plays a central role in cancer progression, not only because of its antioxidant activity but also because it establishes cross-talk with several oncogenic pathways, including Heat Shock Factor1 (HSF1), mammalian target of rapamycin (mTOR), and mutant (mut) p53. Moreover, the involvement of NRF2 in gammaherpesvirus-driven carcinogenesis is particularly interesting. These viruses indeed hijack the NRF2 pathway to sustain the survival of tumor cells in which they establish a latent infection and to avoid a too-high increase of reactive oxygen species (ROS) when these cancer cells undergo treatments that induce viral replication. Interestingly, NRF2 activation may prevent gammaherpesvirus-driven oncogenic transformation, highlighting how manipulating the NRF2 pathway in the different phases of gammaherpesvirus-mediated carcinogenesis may lead to different outcomes. This review will highlight the mechanistic interplay between NRF2 and some oncogenic pathways and its involvement in gammaherpesviruses biology to recapitulate published evidence useful for potential application in cancer therapy.

## 1. Introduction

Nuclear factor erythroid 2-related factor 2 (NRF2) is a transcription factor that protects cells from oxidative stress by regulating various phase II detoxifying/antioxidant enzymes, such as heme oxygenase 1 (HO-1), NAD(P)H quinone oxidoreductase 1 (NQO1), catalase, superoxide dismutase (SOD), and glutathione (GSH) [1]. NRF2 activation must be tightly regulated to sustain cell survival, particularly in cancer cells characterized by a high level of intracellular reactive oxygen species (ROS) responsible for DNA damage induction [2]. ROS increase in cancer cells may be due to the accumulation of dysfunctional mitochondria due to the dysregulation of autophagy, particularly in its selective form, mitophagy [3,4]. Of note, NRF2 plays a key role in regulating several mitochondrial activities, e.g., it increases the mitochondrial membrane potential (ΔΨ) and the availability of substrates for respiration and adenosine triphosphate (ATP) production [5,6]. NRF2 can also increase nicotinamide adenine dinucleotide phosphate (NADPH) by up-regulating gene encoding glucose-6-phosphate dehydrogenase (G6PD), enzymes of the pentose phosphate pathway (PPP), malic enzyme 1 (ME1), and isocitrate dehydrogenase 1 (IDH1) [6].

**Figure 1:**
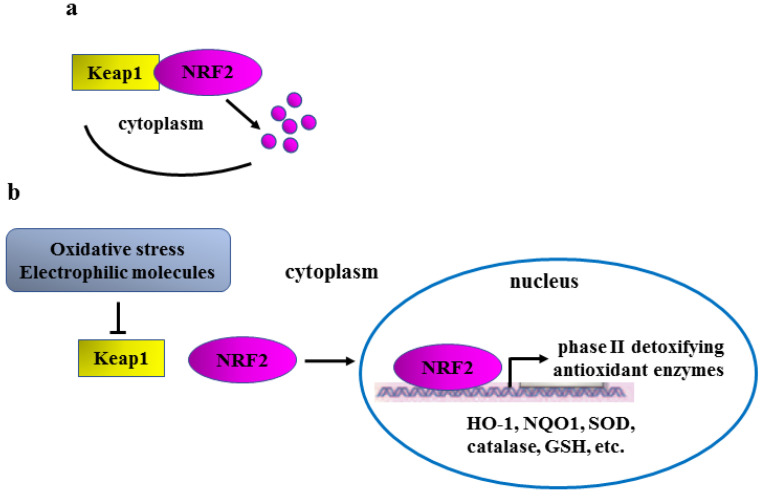
Schematic representation of Keap1/NRF2 regulation. (**a**) In unstressed conditions, Keap1 binds to NRF2, inducing its proteasomal degradation (purple dots). (**b**) Oxidative stress or electrophilic molecules change Keap1 conformation (inverted T sign), impairing Keap1/NRF2 binding (left part); NRF2 is therefore stabilized and can translocate into the nucleus to activate the transcription of phase II detoxifying/antioxidant genes (right part).

NRF2 activation is tightly regulated by Kelch-like ECH-associated protein 1 (Keap1), an ubiquitin ligase that, in unstressed conditions, interacts with NRF2 triggering its proteasomal degradation in the cytoplasm [7] (Figure 1a). Oxidative stress and/or electrophilic molecules, such as plant-derived phenolic compounds, modify cysteine residues 15, 16, and 17 within Keap1 protein, changing its conformation and thus preventing its binding to NRF2 for proteasomal degradation (Figure 1b, left part). Following this “canonical activation”, NRF2 is stabilized and can then translocate into the nucleus to induce the transcription of phase II detoxifying/antioxidant target genes, including HO-1, NQO1, catalase, SOD, and GSH (Figure 1b, right part), to restore cellular redox homeostasis [8].

**Figure 2:**
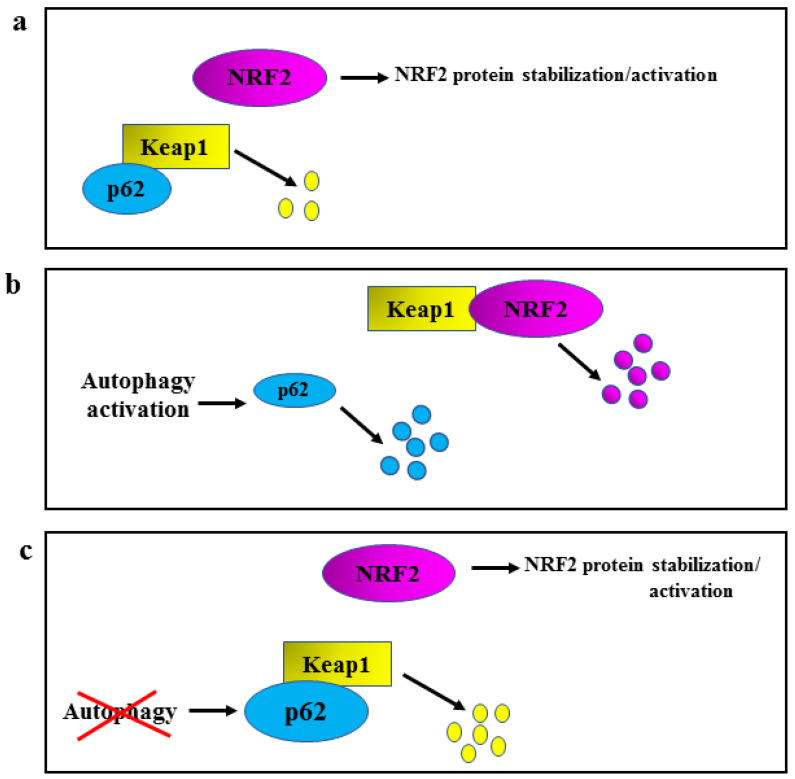
Schematic representation of autophagy/p62/Keap1/NRF2 regulation. (**a**) p62/SQSTM1, herein shortened as p62, triggers a basal Keap1 proteasomal degradation (yellow dots) controlling NRF2 stabilization/activation. (**b**) Autophagy activation degrades p62 (left part, blue dots), stabilizing Keap1 that can bind NRF2 and induce NRF2 degradation (right part, purple dots). (**c**) When autophagy is compromised (red cross), p62 accumulates and can bind Keap1 and trigger Keap1 proteasomal degradation (yellow dots), inducing the stabilization of NRF2.

Another important molecule involved in NRF2 regulation is p62/*sequestosome* 1 (SQSTM1), which induces a “non-canonical stabilization/activation” of NRF2 by triggering Keap1 proteasomal degradation [9,10,11] (Figure 2a). Such regulation of NRF2 intervenes with autophagy, whose activation promotes p62/SQSTM1 degradation [9,10,11] (Figure 2b, left part). Indeed, the reduction of p62/SQSTM1 (degraded through autophagy) keeps Keap1 stabilized so it can bind NRF2 and induce NRF2 degradation (Figure 2b, right part); on the other hand, when autophagy is compromised by several means, p62/SQSTM1 accumulates and binds Keap1 triggering Keap1 proteasomal degradation (Figure 2c, lower part), and inducing the stabilization of NRF2 (Figure 2c, upper part) [9,10,11]. In this manner, the stabilization of NRF2 results as a compensatory mechanism to limit ROS increase due to autophagy dysregulation.

Similarly to p62, p21(Cip1/WAF1) (target of oncosuppressor p53) can bind to Keap1 and interrupt the Keap1/NRF2 interaction, activating NRF2 [12]. Moreover, a direct interaction between NRF2 and p21(Cip1/WAF1) has been also reported [12]. Post-translational modifications, such as phosphorylation, may also regulate NRF2 nuclear translocation. Intriguingly, phosphorylation may influence NRF2 activity both positively and negatively, depending on the residues that undergo phosphorylation and on the kinases that mediate this process. For example, protein kinase C (PKC) may positively influence NRF2 activity [13], while glycogen synthase kinase 3β (GSK-3β) inhibits it [14], underscoring the complex mechanisms involved in the regulation of NRF2 activity.

## 2. NRF2 and Oncogenic Pathways

Another fundamental function of NRF2 is to counteract inflammation that, along with ROS detoxification, plays a key role in preventing carcinogenesis, as evidenced by several studies performed in vitro and in vivo in animal models [15]. The role of NRF2 in inflammation and carcinogenesis mostly depends on its interaction with several molecular pathways. One of the most relevant pathways interacting with NRF2 is represented by heat shock factor 1 (HSF1), the master regulator of the heat shock response. HSF1 and NRF2 control overlapping target genes such as heat shock protein (HSP)70 and p62/SQSTM1 [16,17]. HSP70 plays a crucial role in the folding of proteins involved in key processes, such as the DNA damage response (DDR) of both DNA single and double-strand breaks [18]. HSP70 also sustains lysosomal membrane stability. In this regard, we have demonstrated that the pharmacological inhibition of HSP70 triggers necroptotic cell death in lymphoma cells due to the leakage of lysosomal proteases into the cytosol [19]. Of note, HSP70 collaborates with HSP90, another chaperone whose function is crucial for the folding of a variety of proteins, including oncogenes such as c-Myc and mutant p53 (mutp53) [20,21,22,23,24].

**Figure 3:**
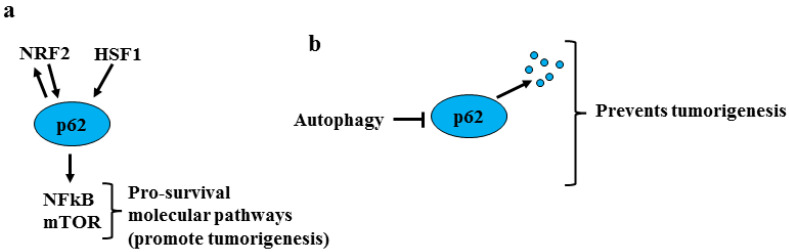
Role of p62/SQSTM1 in tumorigenesis. (**a**) P62/SQSTM1, herein shortened as p62, is a common target of NRF2 and HSF1 activates pro-survival molecular pathways such as NFkB and mTOR that promote tumorigenesis. (**b**) The degradation of p62 by autophagy activation counteracts the first phases of tumorigenesis.

The other common target of NRF2 and HSF1 is p62/SQSTM1, known to play a pro-tumorigenic role not only because it promotes NRF2 stabilization [9] but also because it activates pro-survival molecular pathways such as nuclear factor kappa B (NFkB) and mammalian target of rapamycin (mTOR) [25] (Figure 3a). It has been suggested that autophagy suppresses tumorigenesis by eliminating p62/SQSTM1 [26] (Figure 3b). Interestingly, it has been shown that the reduction of p62/SQSTM1 and its effect on NRF2 may increase ROS levels and the release of inflammatory cytokines by stromal fibroblasts, promoting epithelial cell carcinogenesis [27]. Thus, it is known that oxidative stress induces DNA damage and cancer onset and that long-lasting chronic inflammation may favor all steps of carcinogenesis [28]. Hence, NRF2 transient activation is considered to be mainly cytoprotective during the first phases of carcinogenesis because it limits both DNA damage and inflammation.

Another important pathway that may directly interact with NRF2 is the phosphatidylinositol 3-kinase (PI3K)/AKT/mTOR, which is involved in regulating a variety of vital cellular processes [29]. This pathway has been reported to regulate NRF2 positively and negatively, depending on the cellular context [30]. Although further investigations will better clarify the relationship between NRF2 and mTOR, it has been reported that mutations in the *Nrf2* gene increase the susceptibility of cancer cells to the cytotoxic effects of mTOR inhibitors [31]. Regarding the interplay between NRF2 and mTOR, we have recently shown that the mTOR/p-4EBP1 axis is hyper-phosphorylated following NRF2 activation by Dimethyl fumarate (DMF) [32], a molecule that induces the succination and inactivation of KEAP1 [33]. The activation of mTOR represents a mechanism of resistance of primary effusion lymphoma (PEL) cells undergoing DMF treatment, even if this molecule can still impair PEL survival. Indeed, DMF treatment induces de-phosphorylation and, therefore, inactivation of signal transducers and activators of transcription 3 (STAT3), a pro-survival transcription factor constitutively activated in PEL [32,34]. The cytotoxic effect of DMF against PEL correlates with ROS reduction; hence the traditional knowledge that ROS are only harmful by-products of respiration is being replaced by the finding that they are also important signaling molecules able to sustain oncogenic pathways [35]. In addition, we also found that DMF increases phosphorylation of extracellular signal-regulated kinase (ERK) 1/2, which represents another mechanism of resistance to the treatment due to the induction of pro-survival autophagy [32]. Furthermore, NRF2 inhibition by Brusatol, a quassinoid extracted from *Brucea javanica* able to interfere with NRF2-mediated defense mechanisms [36,37], can restore cancer cell chemosensitivity [38,39]. This indicates that a fine regulation of NRF2 activation is required to balance cancer cell survival/death outcomes [40].

As anticipated above, a key role in NRF2 regulation is played by its phosphorylation mediated by several kinases, whose activation may be influenced, in turn, by NRF2, in feedback loops. Among those kinases, there are the above-mentioned PI3K/AKT and ERK1/2, but also protein kinase C (PKC) [13], c-jun N-terminal kinase (JNK) [41], and p38 MAPK [42]. Interestingly, the activation of NRF2 can negatively influence nuclear factor kappa B (NFkB), the master regulator of cytokines transcription [43]. This may be one of the mechanisms through which NRF2 counteracts the production of pro-inflammatory molecules, including IL6, IL1β, TNFα, COX2, and iNOS. Even if NRF2 and STAT3 [44] can oppositely regulate the initial steps of tumorigenesis, they may synergize to sustain tumor progression, as reported in the case of breast cancer [45], for example. Interestingly, both STAT3 and NRF2 can be phosphorylated by PERK (protein kinase R-like ER kinase) either directly or indirectly through the phosphorylation of GSK3β during the activation of the unfolded protein response (UPR) [46]. UPR is a protective mechanism that helps cells cope with endoplasmic reticulum /ER stress [47]. However, while the direct phosphorylation of NRF2 by PERK results in NRF2 activation, the one mediated by GSK3β inhibits NRF2 [46]. Notably, although UPR is mainly a cell adaptive response to stress, a delicate balance of the activation of its three sensors, namely PERK, IRE1 (inositol-requiring enzyme 1) alpha, and ATF6 (cyclic AMP-dependent transcription factor-6), dictates the final cell death/survival outcome [48]. This decision is also influenced by the pathways activated/inhibited downstream of the UPR sensors, which include NRF2 [47].

## 3. Interplay between NRF2 and p53

**Figure 4:**
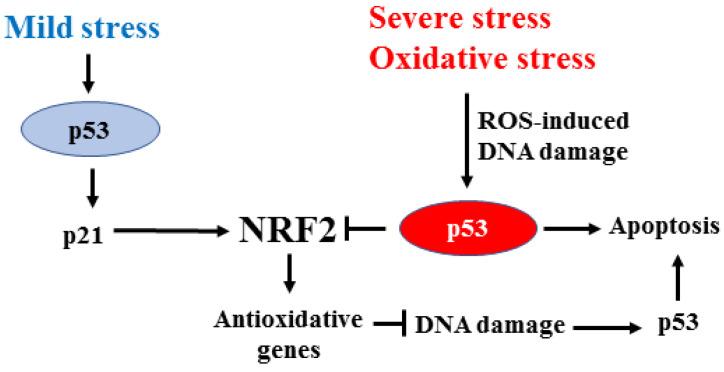
Interplay between p53 and NRF2. In response to mild stress, wtp53 is activated to mainly induce p21 (instead of apoptotic genes) that contributes to NRF2 stabilization. The antioxidant function of NRF2 protects cells from the DNA damage that usually triggers p53 apoptotic activation. Severe oxidative stress, with elevated ROS levels, activates wtp53 apoptotic function and represses the transcription of NRF2 target genes. The mild/severe stress balances the final NRF2/p53 dependent cell survival/death decision in a regulatory feedback loop.

NRF2 may cross-talk with wild-type (wt) and mutant (mut)p53, inhibiting the wtp53 oncosuppressor functions and strengthening the mutp53 oncogenic functions. Both effects contribute to tumor progression and cancer cell resistance to the cytotoxic effects of anticancer therapies. The oncosuppressor p53 is the sensor of DNA damage that activates target genes involved in cell cycle arrest, senescence, or apoptosis, according to the extent of genotoxic damage [49,50]. In particular, high-intensity DNA damage mainly promotes wtp53 apoptotic function [51,52], and the impairment of apoptosis results in the loss of efficacy of cytotoxic therapies [53]. Interestingly, NRF2 and wtp53 share similarities in regulating the redox state, and they may also control each other [54]. Mild stress mainly activates wtp53 to induce p21, which may contribute to NRF2 stabilization [12], leading to cell protection from ROS-induced DNA damage [55]. On the other hand, high-intensity DNA damage, such as the oxidative stress, by inducing p53 post-translational modifications, specifically triggers wtp53 apoptotic activity [50,51,52] along with repression of NRF2 target genes, including x-CT, NQO1, and GST [56]. In this manner, p53/NRF2 interplay may balance the cell’s death/survival decision based on mild/severe stress (Figure 4).

We have shown that NRF2 activation, by, for instance, high glucose or natural compounds (e.g., sulforaphane or curcumin), may reduce p53 apoptotic function [24,38,57,58]. This outcome depends on the inhibition of homeodomain-interacting protein kinase 2 (HIPK2) [39,59] that specifically phosphorylates p53 at Ser46 for apoptotic activation [60]. NRF2, by counteracting oxidative stress, reduces the extent of DNA damage responsible for HIPK2 activation [61]. This outcome impairs the HIPK2/p53 pro-apoptotic activation in favor of the transcription of p21 that, in turn, sustains NRF2 activation [11]. The interplay between NRF2 and HIPK2 is quite intricate and still not completely unveiled. NRF2 may induce HIPK2 gene transcription [62]. The NRF2-induced HIPK2 protein undergoes post-transcriptional modifications by the redox state, leading to the transcription of several antioxidant genes in common with NRF2 (e.g., NQO1, HO-1), thus engaging a pro-survival cross-talk with NRF2 to the detriment of HIPK2 apoptotic activity [62]. NRF2 might also modulate HIPK2 indirectly, at the protein level, by favoring its proteasomal degradation [63,64]. Interestingly, HIPK2 protein regulation may influence its kinase and transcriptional activity and have a different impact on several biological processes [65,66,67,68,69]. In some conditions, HIPK2 may preferentially trigger the transcription of antioxidant genes and support the NRF2-mediated cytoprotective response instead of inducing p53 apoptotic activity, although this hypothesis still needs to be clarified. Therefore, a better understanding of the interplay between the NRF2 and HIPK2/wtp53 pathway could help to elucidate the pro-survival/apoptotic outcome in cancer, especially in the course of anticancer therapies. Since the NRF2 detoxifying activity is important in cancer prevention, cancer cells can hijack this protective mechanism to promote tumor progression [70]. In this regard, NRF2 inhibition could be a valuable strategy for efficiently reestablishing wtp53 apoptotic activity.

Besides being deregulated at the protein level, p53 is inactivated by gene mutations in almost 50% of all types of tumors; those missense mutations often diminish the p53 ability to bind specific DNA recognition sequences of wild-type target genes, losing their oncosupressor function [71]. Mutp53 proteins may sequester various tumor suppressors, including p53 itself (dominant-negative function) and the family members p63 and p73, inhibiting their oncosuppressor functions [72]. The characteristic of mutp53 proteins is their stabilization and increased expression that may depend on binding to cellular chaperones, including HSP/70 and HSP90 [73]. Although mutp53 proteins do not bind to canonical target gene promoters, some of them (i.e., R175H and R273H mutants) may still affect gene transcription by interacting with other transcription factors; this interaction enhances the oncogenic gain of function (GOF) of mutp53. Including increased cell proliferation, migration, and invasion, which contribute to various stages of tumor progression and cancer resistance to therapies [71,73,74,75].

Among the several oncogenic transcription factors that interact with mutp53 to promote cancer progression [76] is NRF2, although molecular interplay still needs to be fully elucidated. Mutp53 has been shown to induce a bi-directional NRF2 target regulation by repressing and activating the NRF2-dependent oxidative stress response [77,78]. Interestingly, in breast cancer cells, mutp53 interacts at the protein level with NRF2, tuning its activity to selectively transcribe genes that sustain cancer cell survival under oxidative stress, such as thioredoxin (TXN) and proteasome system (PSM), and repressing others such as heme oxygenase 1 (HMOX1) [79]. The interplay between mutp53 and NRF2 contributes to the increased survival of cancer cells under oxidative stress by, for instance, exploiting the thioredoxin system. TXN is associated with poor prognosis in breast cancer patients. Thus, a combination therapy that inhibits both TXN and mutp53 may synergistically reduce breast cancer cell growth [79]. The oncogenic interplay between NRF2 and mutp53 has been nicely demonstrated in vivo where, in a K-ras/p53 double mutant mouse model, *Nrf2* depletion decreases pancreatic carcinogenesis and cancer invasion [80]. In another study, five mutp53 proteins have been shown to cooperate with NRF2 to induce the transcription of 26S proteasome and immunoproteasome genes in triple-negative breast cancer (TNBC) cell lines [81]. This cooperation enhances the degradation of tumor suppressor proteins and confers resistance to proteasome inhibitor therapy [81]. In addition, we have demonstrated that mutp53/HSP90 interaction activates a feedback loop between NRF2 and p62 that induces cancer chemoresistance in both pancreatic and breast cancer cells [22,24,39]. These findings suggest that a deeper understanding of the mutp53/NRF2 relationship may pave the way to new more efficient anticancer strategies.

**Figure 5:**
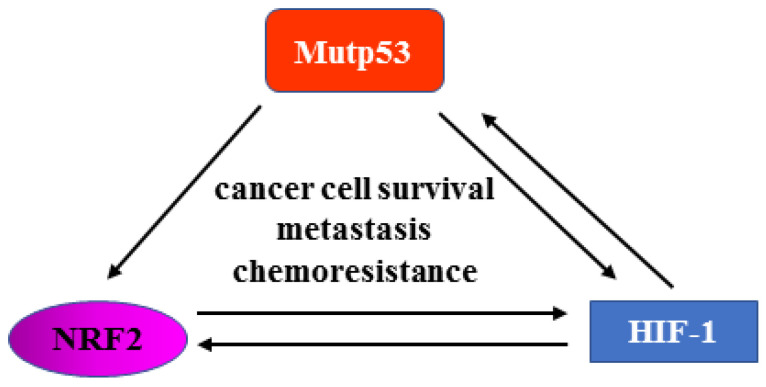
Reciprocal control between mutp53, NRF2, and HIF-1. Mutp53 can sustain NRF2 and HIF-1 activity. NRF2 and HIF-1 can control each other, as well as HIF-1 and mut-p53. The interplay among the oncogenic pathways contributes to tumor progression, metastasis, and chemoresistance.

Hypoxia is a hallmark of solid tumors, and it is responsible for activating hypoxia-inducible factor-1 (HIF-1) oncogenic signaling to promote tumor progression, invasion, and chemoresistance [82]. There is an interplay between hypoxia and mutp53. Hypoxia sustains mutp53 activity, and mutp53 may induce the transcriptional activity of HIF-1 by stimulating the stability of the oxygen-dependent component of the HIF-1 transcription factor, that is, HIF-1alpha [83]. Besides HIF-1, tumor hypoxia activates NRF2 signaling to promote cancer survival, metastasis, and chemoresistance [64]. However, the two signaling pathways can also interact. Thus, NRF2 signaling can activate the HIF-1 response by, for instance, the activation of thioredoxin, though, on the other hand, HIF-1 signaling has been shown to increase NRF2 activation [64]. We can therefore picture a mechanistic interplay among mutp53, NRF2, and HIF-1 to sustain their oncogenic functions and promote tumor progression, invasion, and chemoresistance (Figure 5). Thus, it is conceivable that blocking one oncogenic pathway may influence other pathways and that synchronously blocking them may have greater success in anticancer therapy. For instance, mutp53 can be targeted at the protein level by inhibiting its binding to chaperone [73] or by inducing its autophagic degradation, as demonstrated by our study [84]. However, the interplay between mutp53 and autophagy is quite complex and context-dependent and needs further understanding to shape effective anticancer strategies [85].

Besides mutp53, other oncogenes have been reported to affect NRF2 activity, e.g., Myc has been shown to activate NRF2 and induce tumorigenesis in cells undergoing carcinogenic treatment [86]. Interestingly, c-Myc can establish an interplay with wtp53 as well as with mutp53, inhibiting the first [87] while sustaining the latter to promote pancreatic cancer cell survival [88].

## 4. NRF2 and Gammaherpesvirus-Driven Cancers

We have recently shown in vitro that the silencing of p62/SQSTM1 and NRF2 can counteract the Epstein-Barr virus (EBV)-driven B lymphocyte immortalization [89]. This effect correlates with the reduction of ROS, the main cause of DNA mutations, and the down-regulation of ATM. This kinase is essential to sense DNA damage and to trigger the DDR cascade in response to double-strand DNA breaks. This initiates DNA repair and helps to prevent the accumulation of DNA mutations. NRF2 or p62 /SQSTM1 knockdown also induces the downregulation of H2A histone family member X (H2AX) [89], another key player in DNA repair. Our findings agree with a previous study reporting that H2AX is degraded in oxidative stress conditions caused by a deficient antioxidant response [90]. NRF2 can also be regulated by the other human gammaherpesvirus [91], namely Kaposi’s sarcoma-associated herpesvirus (KSHV). This effect has been observed during de novo infection of human endothelial cells [92] or in naturally infected lymphoma cells [93]. In both cases, NRF2 activation positively regulates viral infection and cell survival/proliferation of virally infected cells.

Regarding gammaherpesvirus-driven carcinogenesis, it has been shown that, besides latent antigens, proteins expressed during the activation of the lytic cycle may contribute to cancer onset [94]. This is mainly because viral lytic proteins promote inflammation that, as said above, is strictly linked to carcinogenesis. NRF2 plays an important role in promoting viral replication, as indicated by the fact that the inhibition of NRF2 reduces the KSHV lytic cycle [93]. Interestingly, the NRF2 target HSP70 [15] is also indispensable for KSHV replication [95]. We have previously shown that the increase of reactive oxygen species (ROS), though it promotes the reactivation of KSHV from latency in lymphoma cells, must be moderate to allow viral replication [96]. Therefore, NRF2 activation is required to prevent too high an increase in ROS level that could promote cell death before viral particles are released [96]. This was observed in cells treated with phorbol diester 12-0-tetradecanoylphorbol-13-acetate (TPA), classic viral lytic cycle inducers that can exert a strong cytotoxic effect against gammaherpesvirus-harboring lymphoma cells [96]. It is also important to consider that EBV-induced NRF2 activation in monocytic cells contributes to the immune escape, which could indirectly facilitate cancer onset. We have found that EBV stabilizes NRF2 in these cells through autophagy inhibition and p62/SQSTM1 accumulation. The reduction of intracellular ROS, consequent to NRF2 activation, impairs the in vitro differentiation of monocytes into dendritic cells (DCs) [97]. Of note, DCs play a pivotal role in initiating the immune response toward new antigens, including viral or tumor antigens. Therefore, reducing their formation has a strong impact on immune response and viral immune escape.

## 5. Conclusions

**Figure 6:**
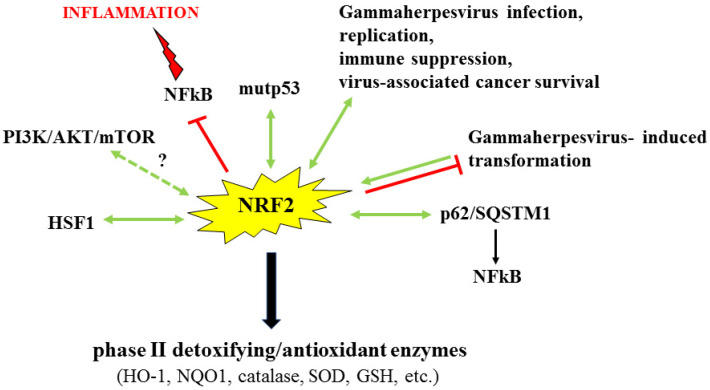
Interplay between NRF2 and oncogenic, pro-survival/inflammatory pathways. Not applicable Schematic representation of the interaction between NRF2 and several pathways that can control each other in a feedback loop. The antioxidant activity of NRF2 and its interaction with those pathways can have a key role in tumor progression.

This update on the key role of NRF2 in cancer survival, progression, and chemoresistance highlights how several of its induced effects rely on the collaboration of NRF2 with oncogenic pathways, such as HSF1, mutp53, and mTOR, and with pro-tumorigenic molecules, such as p62/SQSTM1, sometimes in a feedback loop. Of note, we also highlight how NRF2 activation is exploited by gammaherpesvirus-driven carcinogenesis and immune suppression (Figure 6).

Interestingly, the NRF2 detoxifying activity, hijacked by cancer cells as a protective mechanism, particularly in the course of anti-cancer treatments, is also important in cancer prevention. Therefore, NRF2 manipulation could be a valuable strategy both to prevent cancer and to inhibit its progression, e.g., through the restoration of wtp53 apoptotic activity. In addition, given the key role of NRF2 in gammaherpesvirus-driven carcinogenesis, targeting NRF2 could represent a promising strategy to counteract cancers associated with them. Indeed, NRF2 manipulation may help to reduce the capability of these viruses to infect target cells, to prevent the transformation of cells from which their associated cancers arise, to counteract viral lytic antigen expression and the inflammation that they promote, and, last but not least, to interfere with their-induced immunosuppression. Of note, in cancers associated with gammaherpesviruses, both NRF2 inhibition and NRF2 activation may represent a promising therapeutic approach, as ROS, unless they are too high, can sustain pro-survival, oncogenic pathways, such as STAT3 [32,98,99,100,101]. In light of these findings, future studies should be directed to a better understanding of NRF2 biology, its interaction with oncogenic pathways, and the role of this intricate cross-talk in the different steps of carcinogenesis to target them properly.

## Data Availability

Not applicable.
